# Prevalence and Determinants of Coronary Artery Calcification in Adults With Metabolic Syndrome: A Systematic Review and Meta‐Analysis

**DOI:** 10.1002/clc.70156

**Published:** 2025-06-02

**Authors:** Aftab Ullah, Asif Jan, Hasan Naeem Kareem, Wahby Mohammed Ahmed Babaresh, Abdur Rahim, Syed Shaukat Ali, Waheed Ali Shah, Salim K. Hajwal, Alaa Hamza Hermis, Mustafa Kareem Jawad, Sajjad Sadeq Salman, Murtadha Abdulridha Ajel, Fatimah Saleh Alsuwayidi, Fadhilah N. Alobaidan, Ameer Hasan Kadhem

**Affiliations:** ^1^ Department of Pharmacy University of Peshawar Peshawar Pakistan; ^2^ Department of Pharmacy Abasyn University Peshawar Peshawar Pakistan; ^3^ District Headquarter Hospital (DHQH), Charsadda Charsadda Pakistan; ^4^ Nursing College Al‐Qadisiyah University Al‐Diwaniya Iraq; ^5^ Faculty of Pharmacy University of Aden Aden Yemen; ^6^ Department of Pharmacy CECOS University Peshawar Pakistan; ^7^ Department of Pharmacy University of Malakand Chakdara Pakistan; ^8^ Nursing College Al‐Mustaqbal University Hillah Babil Iraq; ^9^ Nursing College Al‐Mustafa University Baghdad Iraq; ^10^ Nursing Department Kut University College Kut Iraq; ^11^ Armed Forces Hospitals‐Taif Taif Saudi Arabia; ^12^ Royal Commission Health Jubail Saudi Arabia; ^13^ Department of Nursing Al‐Zahrawi University College Karbala Iraq

**Keywords:** cardiovascular risk, coronary artery calcification, meta‐analysis, metabolic syndrome, prevalence

## Abstract

**Background:**

Metabolic syndrome (MetS) is a recognized risk factor for coronary artery calcification (CAC), a subclinical marker of atherosclerosis associated with elevated cardiovascular risk. However, the prevalence and determinants of CAC in individuals with MetS have not been comprehensively synthesized. This systematic review and meta‐analysis aimed to estimate the pooled prevalence of CAC and identify associated factors among adults with MetS.

**Methods:**

A comprehensive search was conducted in PubMed, LILACS, Web of Science, Embase, Scopus, AJOL, and gray literature through December 2024, following PRISMA 2020 guidelines. Eligible studies included adults (≥ 18 years) with MetS, defined by established criteria, and reported CAC scores via validated CT imaging techniques. Observational studies and RCTs were included. Study quality was assessed using the Joanna Briggs Institute checklist. Pooled estimates were derived using a random‐effects model, and heterogeneity was assessed with the *I* ^2^ statistic.

**Results:**

In total, 17 studies comprising 20 745 individuals were included. The pooled prevalence of CAC in adults with MetS was 39.8% (95% CI: 28.4%–52.5%), with wide variation across study design, geography, and imaging modality. Males had a higher CAC prevalence (RR: 2.00), and MetS was linked to increased CAC scores (SMD: 0.10) and odds of calcification (OR: 1.34–1.50). Subgroup analyses showed variability by region and CT modality. High CAC scores were associated with elevated cardiovascular event rates.

**Conclusion:**

CAC affects ~40% of adults with MetS and is associated with higher cardiovascular risk. These findings support the integration of CAC screening in MetS management strategies.

## Summary

- What is currently known about this topic?
  Metabolic syndrome (MetS) increases cardiovascular risk.
  Coronary artery calcium (CAC) is a key marker of subclinical atherosclerosis.
  CAC prevalence varies by sex, region, and imaging modality.
- What is the key research question?
  What is the pooled prevalence of CAC in adults with MetS, and what factors influence CAC scores?
- What is new?
  - Pooled CAC prevalence in MetS patients is 42.7%.
  - Males and certain regions (e.g., USA, Serbia) show higher CAC prevalence.
  - Electron beam CT (EBCT) detects CAC more frequently than multislice CT (MSCT).
- How might this study influence clinical practice?
  CAC scoring should be integrated into cardiovascular risk stratification for MetS patients, especially in high‐risk populations.

## Graphical abstract

This systematic review and meta‐analysis quantified the prevalence and determinants of coronary artery calcification (CAC) among adults with metabolic syndrome (MetS). Findings reveal a substantial burden of CAC in this population, with key determinants including age, hypertension, and dyslipidemia. These results underscore the importance of targeted CAC screening to enhance cardiovascular risk stratification in individuals with MetS.

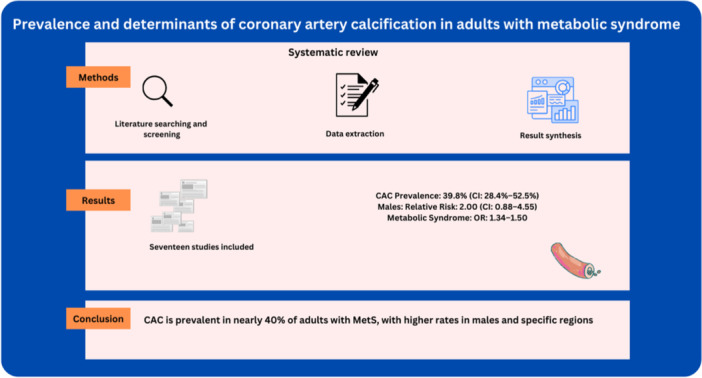

**Abbreviations**
AJOL: African Journals Online
ATP III: Adult Treatment Panel III
BMI: body mass index
CAC: coronary artery calcium
CAD: coronary artery disease
CI: confidence interval
CT: computed tomography
EBCT: electron beam computed tomography
HbA1c: hemoglobin A1c
*I* ^2^
: *I*‐squared (statistical measure of heterogeneity)
IDF: International Diabetes Federation
JBI: Joanna Briggs Institute
LILACS: Latin American and Caribbean Health Sciences Literature
MetS: metabolic syndrome
MSCT: multislice computed tomography
OR: odds ratio
PICO: Population, Intervention, Comparison, Outcome
PRISMA: Preferred Reporting Items for Systematic Review and Meta‐Analysis
PROSPERO: International Prospective Register of Systematic Reviews
RCT: randomized controlled trial
Rayyan: Software for systematic review screening
RevMan: Review Manager (software for meta‐analysis)
WHO: World Health Organization

## 1 Introduction

Metabolic syndrome (MetS), also called syndrome‐X, insulin resistance, is defined by the WHO as a pathologic condition characterized by abdominal obesity, insulin resistance, hypertension, and hyperlipidemia [1]. MetS is associated with the risk of type 2 diabetes, coronary artery disease (CAD), and increased risk of other cardiovascular events [2]. Coronary artery calcium (CAC), a particular feature of coronary atherosclerosis, weakens vasomotor responses and alters atherosclerotic plaque stability, depending on the size and distribution of deposits of CAC [3]. Moreover, CAC is one of the most predictive single cardiovascular risk markers in asymptomatic individuals, capable of adding predictive information beyond the traditional cardiovascular risk factors [4].

The precise biological mechanisms through which MetS contributes to the development and progression of CAC deposition are presently not fully understood or elucidated in the literature. The connection between individual components of MetS, such as hypertension, hyperglycemia, dyslipidemia, and central obesity, and the development of atherosclerosis and calcification is well established in the literature [5]. However, the combined effects and interactions of these risk factors, when considered together, are not yet fully understood.

There is a paucity of data on the prevalence and associated factors of CAC among adults with MetS. Addressing these knowledge gaps will not only enhance our understanding of the interplay between MetS and CAC but also improve risk stratification, prevention, and treatment strategies for cardiovascular diseases in MetS patients [6]. These will also help develop clear guidelines on integrating CAC scores into the clinical management of patients with MetS; including deciding when to use CAC scoring, interpreting the results in the context of MetS, and tailoring interventions based on CAC scores [7].

While the association between MetS and CAD has been well established—particularly through studies focusing on individual components such as hypertension, dyslipidemia, hyperglycemia, and obesity—the specific burden of CAC in individuals with MetS remains insufficiently characterized. Most prior meta‐analyses have centered on broader cardiovascular outcomes or general risk stratification, whereas our review focuses specifically on the prevalence and determinants of CAC in adults with MetS. Given that CAC is a recognized marker of subclinical atherosclerosis and a predictor of adverse cardiovascular events, understanding its distribution in MetS populations is critical. This systematic review and meta‐analysis aimed to (1) estimate the pooled prevalence of CAC among individuals with MetS and (2) identify key determinants influencing CAC scores. By synthesizing data across diverse populations and study designs, our approach addresses methodological heterogeneity and provides a more comprehensive and globally relevant perspective.

## 2 Methods

This systematic review and meta‐analysis was conducted following protocol registration in PROSPERO. The protocol was neither published nor under consideration by any journal. The Preferred Reporting Items for Systematic Review and Meta‐Analysis (PRISMA 2020) method was followed in screening the included studies [8].

### 2.1 Information Sources

The following databases: PubMed, Latin American and Caribbean Health Sciences (LILACS), Medline, Web of Science, Embase, Scopus, and African Journals Online (AJOL) were identified and included. We have also reached gray literature less accessible studies via Google search and other sources. These databases help capture a broad spectrum of literature across different regions, languages, and fields, which is critical for a systematic review that aims to be inclusive, comprehensive, and methodologically sound.

### 2.2 Search Terms

The search terms were developed using the Population, Intervention, Comparison, and Outcomes (PICO) framework. The PICO elements were used as follows: Metabolic syndrome as “population,” computed tomography as “intervention,” and coronary artery calcium scores as “outcome.” However, the element “comparison” was not included as it was not relevant in this review. Each element was further explored to find similar or related terms using the relevant studies' medical subject headings (MeSH) and keywords. Boolean operators OR and AND were used to connect similar and different terms, respectively, as presented in Table 1, showing the search terms used to generate articles for selection as follows: Metabolic Syndrome AND computed tomography AND coronary artery calcium scores. See the search for the PubMed database below:

**Table 1:** Search terms with Boolean operators.

Database	Query	No. of hits
PubMed	(“Coronary Artery Calcification”[MeSH] OR “Coronary Artery Calcium” OR “Coronary Calcium” OR “Arterial Calcification” OR “CAC”) AND (“Metabolic Syndrome”[MeSH] OR “Metabolic Syndrome X” OR “Syndrome X” OR “Insulin Resistance Syndrome” OR “MetS”) AND (“Prevalence”[MeSH] OR “Epidemiology” OR “Risk Factors”[MeSH] OR “Associated Factors” OR “Predictors” OR “Determinants”)	127
Embase	(“coronary artery calcification”/exp OR “coronary artery calcium” OR “coronary calcium” OR “arterial calcification” OR CAC) AND (“metabolic syndrome x”/exp OR “metabolic syndrome” OR “syndrome x” OR “insulin resistance syndrome” OR MetS) AND (“prevalence”/exp OR “epidemiology” OR “risk factor”/exp OR “associated factors” OR “predictors” OR “determinants”)	810
Scopus	TITLE‐ABS‐KEY(“Coronary Artery Calcification” OR “Coronary Artery Calcium” OR “Coronary Calcium” OR “Arterial Calcification” OR CAC) AND TITLE‐ABS‐KEY(“Metabolic Syndrome” OR “Metabolic Syndrome X” OR “Syndrome X” OR “Insulin Resistance Syndrome” OR MetS) AND TITLE‐ABS‐KEY(Prevalence OR Epidemiology OR “Risk Factors” OR “Associated Factors” OR Predictors OR Determinants)	523
Web of Science	TS = (“Coronary Artery Calcification” OR “Coronary Artery Calcium” OR “Coronary Calcium” OR “Arterial Calcification” OR CAC) AND TS = (“Metabolic Syndrome” OR “Metabolic Syndrome X” OR “Syndrome X” OR “Insulin Resistance Syndrome” OR MetS) AND TS = (“Prevalence” OR “Epidemiology” OR “Risk Factors” OR “Associated Factors” OR “Predictors” OR “Determinants”)	484
AJOL	“Coronary Artery Calcification” OR “Coronary Calcium” OR “Arterial Calcification” AND “Metabolic Syndrome” OR “Insulin Resistance Syndrome” OR MetS AND Prevalence OR Risk Factors OR Determinants	25
LILACS	(“Coronary Artery Calcification” OR “Coronary Artery Calcium” OR “Coronary Calcium” OR “Arterial Calcification” OR CAC) AND (“Metabolic Syndrome” OR “Metabolic Syndrome X” OR “Syndrome X” OR “Insulin Resistance Syndrome” OR MetS) AND (“Prevalence” OR “Epidemiology” OR “Risk Factors” OR “Associated Factors” OR “Predictors” OR “Determinants”)	42

### 2.3 Eligibility Criteria

This review included studies on adults (18 years and older) diagnosed with MetS according to recognized criteria (e.g., ATP III, IDF, WHO). Eligible studies clearly defined and measured MetS and its components (e.g., abdominal obesity, hypertension, hyperglycemia, dyslipidemia) and reported CAC scores using validated methods like cardiac CT or electron beam computed tomography (EBCT). Study designs considered included observational studies (e.g., cross‐sectional, cohort, case‐control), randomized controlled trials (RCTs), and case‐control studies, published in peer‐reviewed journals or as gray literature (e.g., conference proceedings, theses), in English, and from database inception to December 2024.

Exclusions applied to studies involving children, adolescents, or populations with conditions that could bias results (e.g., known cardiovascular disease, chronic kidney disease, systemic inflammatory conditions). Studies lacking clear MetS definitions, using outdated criteria, or nonvalidated CAC assessment methods were also excluded, as were case reports, case series, reviews, editorials, and commentaries.

### 2.4 Selection Process

The literature was searched and screened by A.U. and A.J., both of whom have published systematic reviews. Articles retrieved from the databases were imported and screened for duplicates and relevance using Rayyan software. Both researchers independently screened the articles based on the titles and abstracts. Any discrepancies between the two independent reviewers during study selection and data extraction were resolved through discussion and, when necessary, adjudicated by a third senior reviewer to ensure consensus. Subsequently, the full‐text articles were downloaded and screened based on the inclusion and exclusion criteria.

### 2.5 Quality Assessment

The methodological quality of the included studies was assessed using the Joanna Briggs Institute's critical appraisal checklist for prevalence studies [9]. The checklist contains 9 items, and each item was evaluated where correct information had a score of 1, and “no” or “unclear/not applicable” had a score of 0. Studies scoring 1–3 were considered low quality, those scoring 4–6 were classified as moderate quality, and those scoring 7–9 were deemed high quality. Only studies with moderate or high quality were included in the final analysis. Two independent reviewers conducted the quality assessments.

### 2.6 Data Extraction

Information such as (i) authors, (ii) year of publication, (iii) country, (iv) study design (v) sample size, (vi) mean age of the participants, (vii) prevalence of CAD, (viii) CAC quality score, and (ix) method of CAC evaluation from each of the included studies extracted using an Excel spreadsheet.

### 2.7 Synthesis Method

The primary researcher (A. U.) extracted data from the included studies, which was independently verified by a second researcher (I. G.). Heterogeneity was described by reporting variations in study design and population characteristics. Pooled prevalence and risk ratio (RR) estimates—both overall and subgroup‐specific—were calculated using the DerSimonian–Laird random‐effects model. This method accounts for both within‐study and between‐study variability, making it well‐suited for meta‐analyses that involve clinical and methodological diversity. The DerSimonian–Laird method extends the fixed‐effects model by incorporating an estimate of the between‐study variance (*τ* ^2^) into the weighting of individual studies. Instead of weighting studies solely based on their within‐study variance, this approach assigns weights that are inversely proportional to the sum of within‐study and between‐study variance [10]. This allows for more conservative and realistic estimates when there is substantial heterogeneity among studies. Meta‐analyses were conducted using R software (version 4.4), employing the meta and metafor packages [11]. Heterogeneity was assessed using the *I* ^2^ statistic, which quantifies the proportion of variation in effect estimates due to heterogeneity rather than chance [12, 13]. Subgroup analyses were performed based on study design, geographic region, and the type of CT scan used for CAC assessment. Results were reported as pooled estimates with 95% confidence intervals (CIs), and prediction intervals [14] were also provided to reflect the expected range of effects in future similar studies [15].

### 2.8 Reporting Bias Assessment

The presence of publication bias was assessed and presented graphically using a funnel plot in R software. This method ratified the use of funnel plots, estimated the number and outcomes of missing studies, and adjusted the meta‐analysis to incorporate the theoretical missing studies [16]. We set the level of significance at *p* < 0.05 for the Egger's test.

## 3 Results

### 3.1 Study Selection

**Figure 1:**
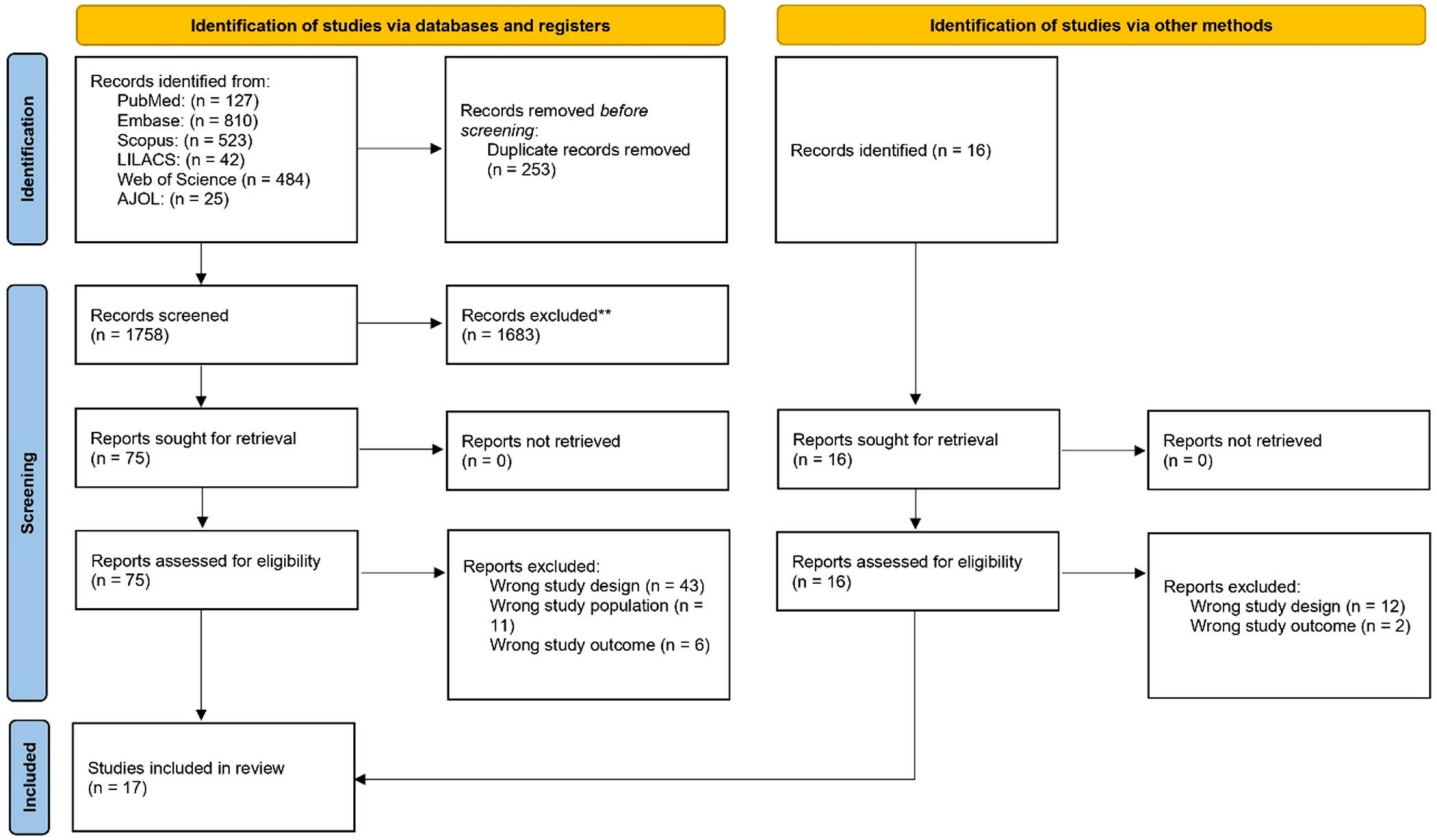
A schematic representation of the flow of studies reviewed in the course of the systematic review and meta‐analysis. Preferred items for systematic review and meta‐analysis (PRISMA 2020). **Excluded due to irrelevant to the research question.

A total of 2011 studies were identified through online databases, free Google searches, and gray literature. After removing duplicates and conducting both the first and second rounds of screening, 17 articles published between 2004 and 2024 were selected for inclusion in the review for quality assessment and evidence synthesis, as illustrated in Figure 1.

### 3.2 Study Characteristics

The cumulative sample size of these studies was 20 745. There was a male preponderance in all the studies. The age distribution of participants ranged from 22 to 82 years with a mean of 51.2 years. Five studies were prospective cohort studies, nine were cross‐sectional; while the remaining four studies were retrospective. The earliest study was conducted by Kulo et al. [17] in 2004, which is a cross‐sectional study. The latest among them is that of Masrouri et al. [18] in 2024, a prospective cohort study in Iran with 2127 patients. The prevalence of CAD from this review ranged from 26% according to Santos et al. [19] (in Brazil) to 66.5% in the USA according to Wong and colleagues (Table 2).

**Table 2:** The basic characteristics of the included studies.

References	Year	Country	Study design	Sample size	Mean age	Males (*n*)	Females (*n*)	Prevalence of CAD	Quality score	Method of CAC evaluation
Wong et al. [20]	2012	USA	Cross‐sectional	279	52.7 ± 9.9	NA	142	58.8	9	Multislice CT
Kullo et al. [17]	2004	USA	Cross‐sectional retrospective	204	47.9 + 14.4	99	105	38%	7	Electron beam CT
Ellison et al. [21]	2005	USA	Cross‐sectional	3166	54.43	1300	1,866	56.30%	7	Multislice CT
Wong et al. [22]	2006	USA	Cross‐sectional	4468		NA	1876	66.50%	7	Electron beam CT
Santos et al. [19]	2007	Brazil	Cross‐sectional	329	46 ± 7	NA	NA	26%	8	Electron beam CT
Ibebuogu et al. [23]	2009	USA	Prospective study	356		NA	NA	43%	8	Electron beam CT
Cao et al. [24]	2013	China	Cross‐sectional retrospective	572	53.3	261	NA	42.50%	7	Multislice CT
Seo et al. [25]	2015	Korea	Cross‐sectional	3037	43.5 + 7.25	2816	NA	24.70%	8	Multislice CT
Lee et al. [26]	2015	Korea	Cross‐sectional retrospective	2220	45.1 ± 9.4	NA	NA	23.2	8	Multislice CT
Lee et al. [27]	2015	Korea	Prospective	887	52.5	746	NA	34.30%	8	Multislice CT
Kim et al. [28]	2016	Korea	Prospective	825	56.6 ± 7.5	486	NA	54.4	8	Multislice CT
Malik et al. [29]	2017	USA	Prospective	1738	62.2 ± 10.2	717	NA	55.20%	9	Electron beam CT
Ekblom‐Bak et al. [30]	2018	Sweeden	Cross‐sectional	181	57.25	NA	NA	47%	8	Multislice CT
Marjanovic et al. [31]	2023	Serbia	Cross‐sectional	100	56.76 ± 6.86	29	NA	49%	8	Multislice CT
Fakhry et al. [32]	2023	Egypt	Cross‐sectional	150	55.42 ± 9.39	131	19	43.10%	6	Multislice CT
Masrouri et al. [18]	2024	Iran	Prospective	2127	48.0 + 4.2	950	NA	29.6	8	Multislice CT
Tangjitgamol et al. [33]	2024	Thailand	Cross‐sectional	106	45	NA	NA	47.2	9	Multislice CT

#### 3.2.1 Assessment of Pooled Prevalence of CAC in Metabolic Syndrome

**Figure 2:**
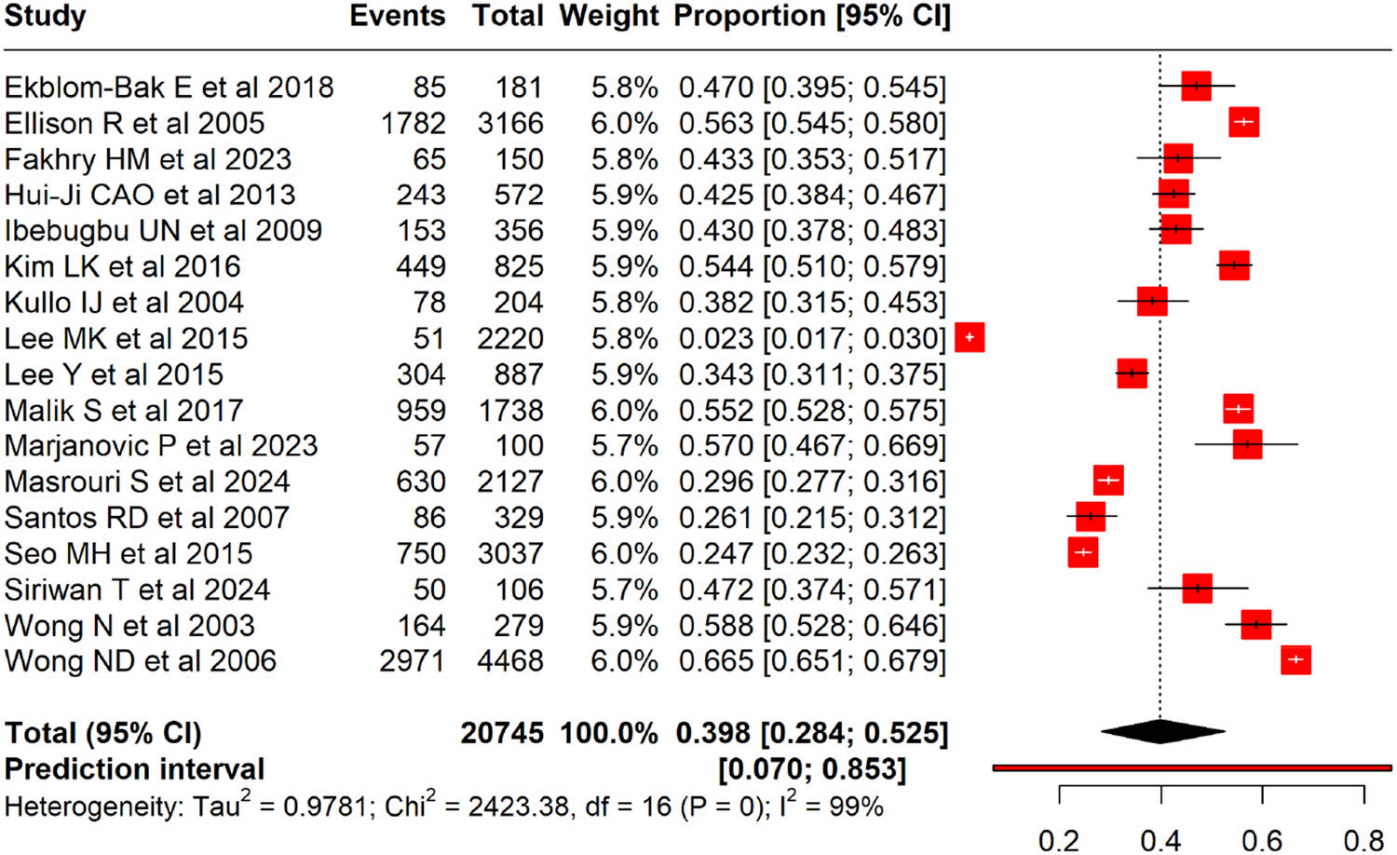
A forest plot showing the pooled prevalence of CAC among adults with metabolic syndrome.

**Figure 3:**
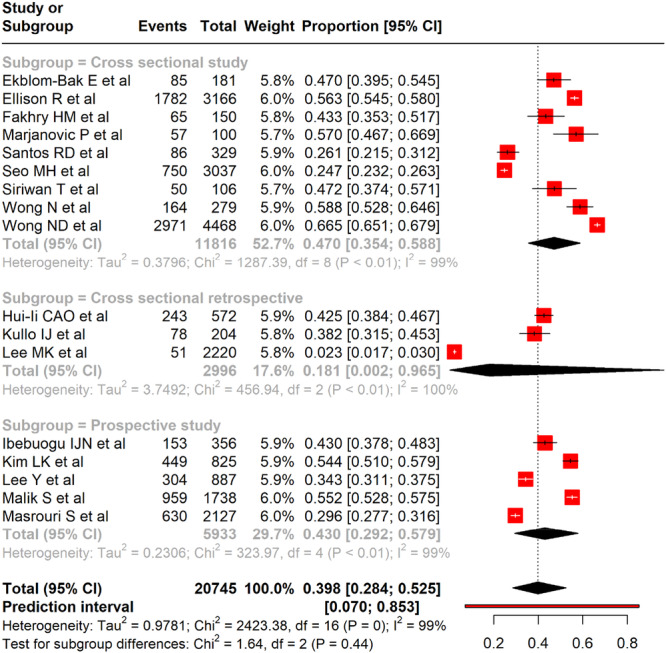
A forest plot showing the pooled prevalence of CAC and subgroup analysis based on study design among adults with metabolic syndrome.

The pooled prevalence of CAC among adults with MetS, based on 17 studies, was 39.8% (95% CI: 28.4%–52.5%), with a wide prediction interval (7.0%–85.3%), indicating substantial variability across study populations (Figure 2). Prevalence ranged from 2.3% (Lee et al. [26]) to 66.5% (Wong et al. [22]). Heterogeneity was considerable (*I* ^2^ = 99%, *p* < 0.001). Subgroup analysis by study design showed that cross‐sectional studies reported a pooled prevalence of 47.0% (95% CI: 35.4%–58.8%), prospective studies 43.0% (95% CI: 29.2%–57.9%), and cross‐sectional retrospective studies 18.1% (95% CI: 0.2%–96.5%). Despite these subgroupings, heterogeneity remained high (*I* ^2^ = 99%) and differences across study designs were not statistically significant (*χ* ^2^ = 1.64, *p* = 0.44) (Figure 3).

**Figure 4:**
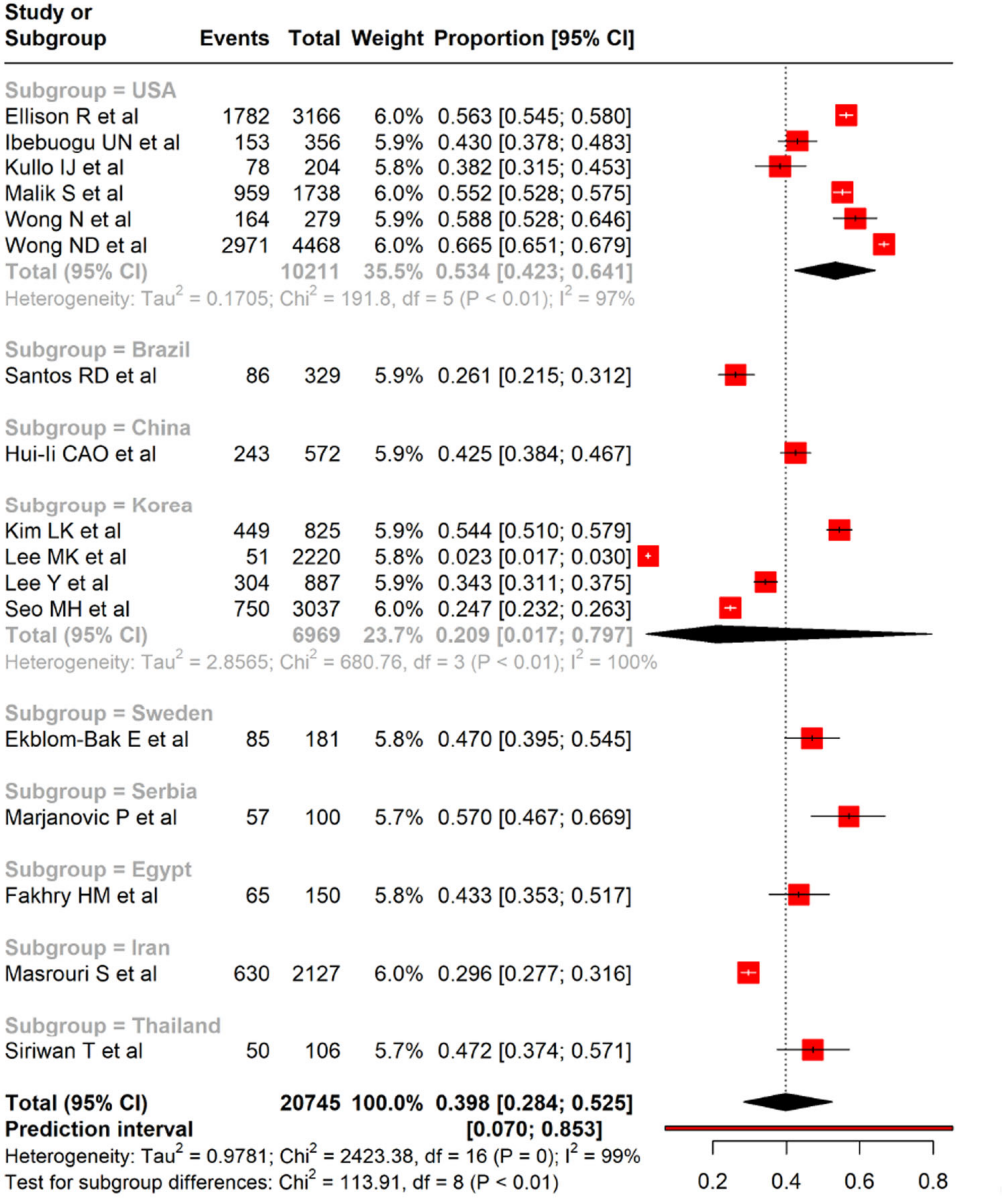
A forest plot showing the pooled prevalence of CAC and subgroup analysis based on study location (country) among adults with metabolic syndrome.

Geographic variation significantly contributed to the heterogeneity in CAC prevalence (Figure 4). Studies from the USA (six studies) showed a higher pooled prevalence of 53.4% (95% CI: 42.3%–64.1%) with moderate heterogeneity (*I* ^2^ = 97%), while Korea (four studies) reported a lower pooled prevalence of 20.9% (95% CI: 1.7%–79.7%) with extreme heterogeneity (*I* ^2^ = 100%). Other countries contributed single studies with varying prevalence estimates: Brazil (26.1%), China (42.5%), Sweden (47.0%), Serbia (57.0%), Egypt (43.3%), Iran (29.6%), and Thailand (47.2%). The test for subgroup differences was significant (*χ* ^2^ = 113.91, df = 8, *p* < 0.01), suggesting that geographic location may partly explain the observed heterogeneity in CAC prevalence among individuals with MetS.

#### 3.2.2 Assessment Methodology and Prevalence of CAC in Metabolic Syndrome

**Figure 5:**
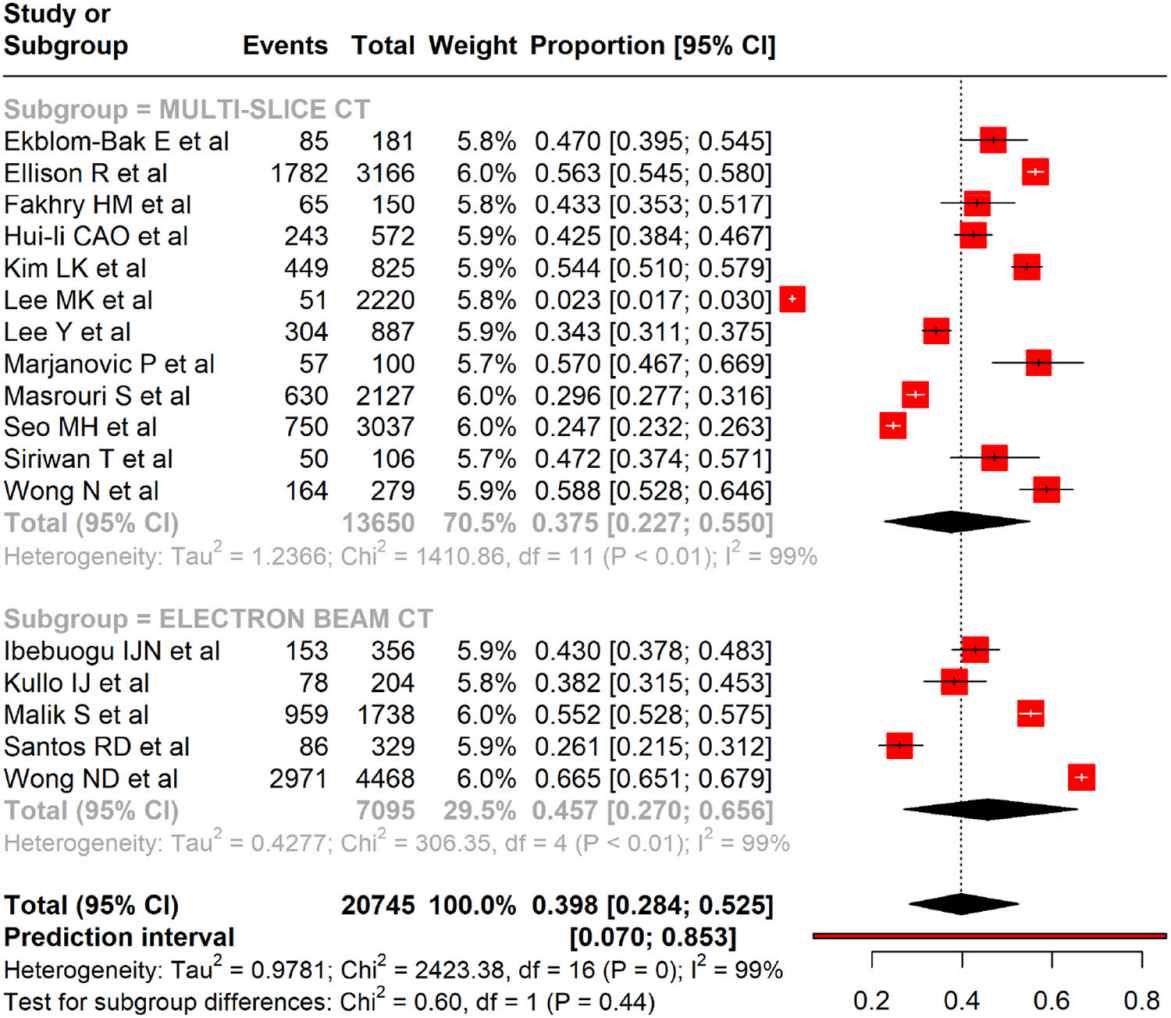
This figure shows a forest plot of the subgroup analysis based on the modality of CAC assessment among subjects with metabolic syndrome.

Across the studies analyzed, two main imaging modalities were used to evaluate CAC: EBCT and multislice computed tomography (MSCT). Of the selected studies, five employed EBCT, while nine used MSCT. According to the forest plot (Figure 5), this subgroup analysis based on CT imaging modality demonstrates variation in the prevalence of CAC in individuals with MetS. Studies using MSCT (12 studies) yielded a pooled prevalence of 37.5% (95% CI: 22.7%–55.0%) with significant heterogeneity (*I* ^2^ = 99%). In comparison, studies using EBCT (5 studies, *n* = 7095) showed a higher pooled prevalence of 45.7% (95% CI: 27.0%–65.6%), also with substantial heterogeneity (*I* ^2^ = 99%).

#### 3.2.3 Reporting Biases

**Figure 6:**
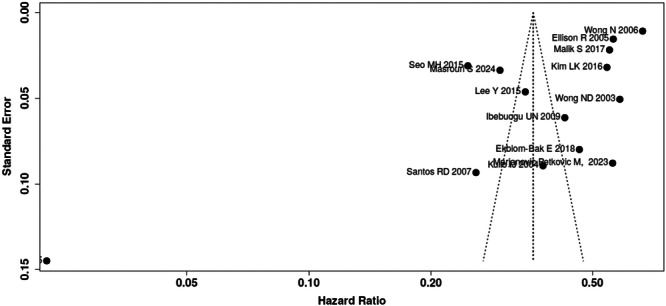
Funnel plot assessing publication bias.

The funnel plot (Figure 6) assessing publication bias showed asymmetry, indicating potential bias in the reporting of CAC prevalence confirmed with the Egger's test (*p* < 0.01). Notably, studies with larger effect sizes predominantly originated from the United States and Europe, whereas studies reporting lower CAC prevalence were largely from Asia. This distribution may point to both regional differences in CAC prevalence and a possible tendency for studies with high prevalence estimates to be more widely published.

#### 3.2.4 Coronary Artery Calcium Scores by Metabolic Syndrome Status

**Figure 7:**
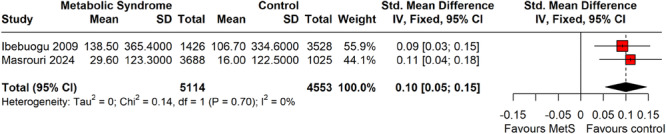
A forest plot of subgroup analysis from selected studies on CAC scores by metabolic syndrome status.

As shown in Figure 7, this forest plot compares CAC scores between individuals with MetS and controls across two studies. The standardized mean differences (SMDs) were 0.09 (95% CI: 0.03–0.15) for Ibebuogu et al. [23] and 0.11 (95% CI: 0.04–0.18) for Masrouri et al. [18], both favoring the MetS group. The overall pooled SMD was 0.10 (95% CI: 0.05–0.15), indicating a small but statistically significant increase in CAC scores among individuals with MetS. Heterogeneity was negligible (*I* ^2^ = 0%, *τ* ^2^ = 0, *p* = 0.70), suggesting consistency between studies.

#### 3.2.5 Sex Differences in CAC Prevalence and Severity

**Figure 8:**
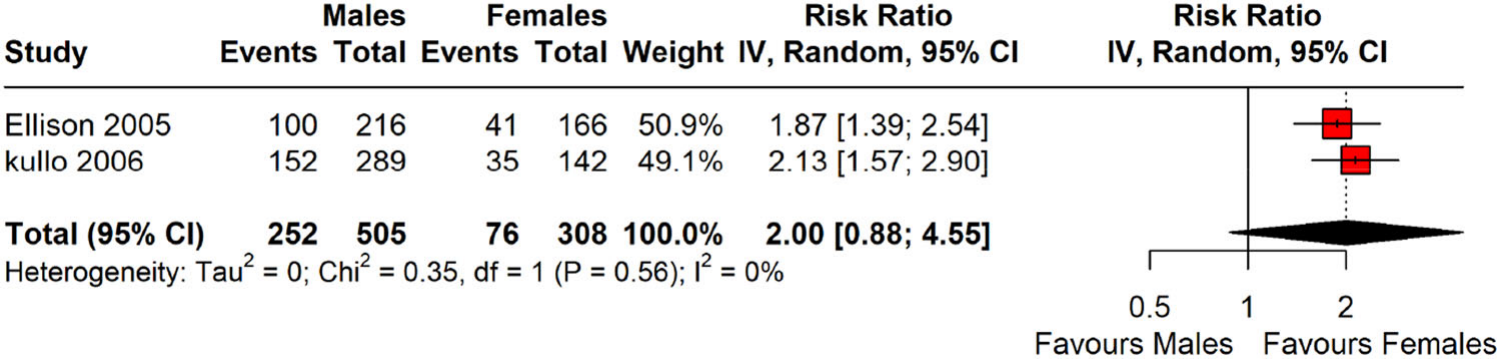
A forest plot showing the sex differences in CAC prevalence and severity.

As illustrated in Figure 8, the sex‐based differences were consistently observed in CAC prevalence. The two included studies—Ellison et al. [21] and Kullo et al. [17]—reported RRs of 1.87 (95% CI: 1.39–2.54) and 2.13 (95% CI: 1.57–2.90), respectively, both indicating a higher prevalence of CAC in males compared to females. The pooled risk ratio was 2.00 (95% CI: 0.88–4.55), suggesting a trend toward increased CAC in males, though not statistically significant due to the wide CI crossing 1. Heterogeneity was absent (*I* ^2^ = 0%, *τ* ^2^ = 0, *p* = 0.56), indicating consistency between the studies.

#### 3.2.6 Multivariate Logistic Regression Analysis of CAC and Metabolic Status

CAC risk was assessed across metabolic health statuses, and adjusted for potential confounders such as age, sex, hypertension, antihypertensive medication, hypercholesterolemia, cholesterol‐lowering medication, and cigarette smoking. Ibebuogu et al. [23] found that individuals with MetS had 1.4‐fold higher odds of any CAC (95% CI, 1.06–2.1; *p* = 0.005) and 1.5‐fold higher odds of having a CAC score above 75% (95% CI, 1.3–1.8; *p* = 0.008) compared to those without MetS. For those with diabetes, the odds ratios were further elevated, reaching 1.6 for any CAC (95% CI, 1.3–2.4; *p* < 0.001) and 3.46 for CAC scores above 100 (95% CI, 1.6–7.4; *p* < 0.001). Similarly, Missouri et al. [18] reported that individuals with MetS had significantly higher odds of any CAC (OR = 1.34, 95% CI, 1.10–1.64; *p* = 0.004) and CAC scores above 100 (OR = 1.50, 95% CI, 1.05–2.15; *p* = 0.028), with even greater risks observed among individuals with diabetes.

#### 3.2.7 Impact of Physical Fitness on CAC Scores in Metabolic Syndrome

CAC prevalence was inversely related to physical fitness levels, as shown in Ekblom‐Bak et al. [30]. Among individuals with MetS, lower fitness levels were associated with higher CAC scores, especially among men, where 48% with low fitness had MetS compared to 9% with high fitness. Furthermore, CAC scores over 100 were more prevalent in low‐fitness individuals, indicating a potential protective effect of physical fitness against coronary calcification.

#### 3.2.8 Cardiovascular Risk and Outcome Data in Persons With CAC

Higher CAC levels were associated with an increased incidence of cardiovascular events across all metabolic health statuses, with the risk escalating significantly in individuals with MetS. Individuals with a CAC score of 0 had the lowest event rate (2.04/1000 person‐years), which rose to 5.98 for CAC scores of 1–99 and 19.44 for CAC > 100. Furthermore, the event rates were higher for each CAC level, starting at 4.88 for CAC = 0, increasing to 7.33 for CAC 1–99, and reaching 25.09 for CAC > 100.

#### 3.2.9 Cardiovascular Events and Mortality Rates by CAC Score

The incidence rate of cardiovascular events was notably higher among individuals with elevated CAC scores and metabolic abnormalities. For instance, in the study by Masrouri et al. [18], the incidence of events among individuals with MetS was 25.09/1000 person‐years for those with CAC scores above 100, in contrast to 7.33/1000 person‐years in those with CAC scores between 1 and 99. Similarly, all‐cause mortality was positively correlated with CAC score severity. Individuals with MetS and CAC > 100 had an incidence rate of 9.06/1000 person‐years, significantly higher than rates observed in those with CAC scores < 100. This trend was also observed in those with diabetes, suggesting a cumulative impact of MetS and coronary calcification on cardiovascular morbidity and mortality.

## 4 Discussion

CAC is a well‐established marker of subclinical atherosclerosis and is increasingly recognized for its prognostic value in cardiovascular risk assessment, particularly in individuals with MetS. Despite CAC scoring being endorsed as a secondary risk stratification tool in cardiovascular care, its application in MetS remains underexplored. This meta‐analysis addresses this gap by estimating the pooled prevalence of CAC and identifying factors influencing its occurrence in MetS populations. These findings may help clinicians identify high‐risk subgroups within MetS who could benefit from CAC screening, especially when conventional risk scores are inconclusive. While our results do not challenge existing guidelines, they provide foundational evidence that could inform future refinements in personalized risk stratification for MetS. Additionally, this review underscores the need for prospective studies to assess how CAC evaluation might influence treatment strategies in MetS populations. By synthesizing data from 17 studies across various regions, this analysis highlights the clinical relevance of CAC assessment in MetS and identifies areas that warrant further investigation. The meta‐analysis forest plot, summarizing data from 17 studies on the prevalence of CAC in adults with MetS, reveals a pooled prevalence of 39.8% (95% CI: 28.4%–52.5%), with considerable variability across studies. The prediction interval ranged from 7.0% to 85.3%, indicating the expected range of true prevalence in future studies of similar design.

The pooled prevalence of CAC among individuals with MetS was 39.8% (95% CI: 28.4%–52.5%), with significant variations based on sex, geographic region, and imaging modality. Males consistently exhibited higher CAC prevalence and severity than females, which could be attributed to sex‐related differences in cardiovascular risk profiles and the pathophysiology of atherosclerosis. Studies from the United States and Serbia reported the highest prevalence rates, suggesting potential contributions of genetic, lifestyle, or environmental factors. In contrast, studies from Asia recorded lower prevalence, suggesting that geographic and possibly genetic or lifestyle factors could influence the progression of coronary calcification in MetS patients.

### 4.1 Implications of CAC Prevalence in MetS

This analysis reaffirms the role of MetS as a significant contributor to coronary calcification and, consequently, cardiovascular risk. Elevated CAC scores demonstrated a robust correlation with heightened risks of adverse cardiovascular events, such as myocardial infarction and stroke, as well as increased overall mortality. This finding highlights the prognostic value of CAC as a marker for subclinical atherosclerosis and cardiovascular risk stratification. The findings also highlight the potential of CAC assessment in tailoring preventive and therapeutic strategies, particularly in resource‐limited settings where traditional risk stratification tools may be less accessible.

### 4.2 Comparative Analysis of Methodologies

The type of CT used for CAC evaluation influenced the reported prevalence rates. EBCT demonstrated slightly higher detection rates compared to MSCT, possibly due to variations in sensitivity and resolution. The marked heterogeneity observed across studies (*I* ^2^ = 99.8%, *p* < 0.001) further highlights the influence of methodological differences, including study designs, CAC scoring systems, and participant characteristics.

### 4.3 Strengths and Limitations

This study adheres to PRISMA guidelines, with rigorous methodological quality assessment and comprehensive subgroup analyses, strengthening its validity. A key strength is the focused analysis presented in Figures 7 and 8, which included only studies with stratified data on CAC scores by metabolic status, enabling meaningful insights into coronary calcification differences between individuals with and without MetS. Although not all studies contributed to these figures, the selected studies provided valuable information on cardiovascular risk differentiation. Subgroup analyses addressing CAC assessment methods (EBCT vs. MDCT), geographic location, and study design further examined sources of heterogeneity, offering a more nuanced interpretation of pooled prevalence estimates. This approach underscores the importance of methodological consistency in future research on CAC in MetS populations. Additionally, updated visualizations aim to assist clinicians in integrating CAC scoring into risk assessment and management strategies.

However, there are notable limitations. The heterogeneity observed in the meta‐analysis and the varying degrees of multivariate adjustment in the included studies hindered our ability to conduct a pooled analysis accounting for potential collinearity among MetS components like diabetes, hypertension, and dyslipidemia. As these factors are often interrelated, caution is needed when interpreting their independent associations with CAC. Furthermore, the lack of stratified data on high versus low CAC scores limited our ability to conduct detailed subgroup analyses based on CAC severity, restricting our understanding of how varying degrees of calcification may influence cardiovascular risk. The observed funnel plot asymmetry suggests the possibility of publication bias, including small‐study effects or selective reporting, which could impact the robustness of our findings. Exclusion of non‐English studies and reliance on observational data may also limit the generalizability of the conclusions. Future studies should aim for standardized multivariable models, consistent reporting of CAC severity, and consideration of publication bias to refine risk stratification and clinical decision‐making in MetS patients.

## 5 Conclusion

This systematic review underscores the substantial burden of CAC among individuals with MetS and its pivotal role in cardiovascular risk stratification. The findings reveal that males with MetS exhibit disproportionately higher CAC levels, suggesting the need for gender‐sensitive approaches in cardiovascular risk assessment. Furthermore, the heightened cardiovascular risks associated with elevated CAC levels emphasize the utility of CAC as a critical tool for early detection and risk management. Integrating routine CAC evaluation into global health strategies, particularly for high‐risk populations in regions with a high MetS prevalence, holds the potential to enhance the prevention and management of cardiovascular diseases worldwide.

## Author Contributions

Conceptualization: Aftab Ullah, Asif Jan, Hasan Naeem Kareem, and Wahby Mohammed Ahmed Babaresh. Data curation: Syed Shaukat Ali, Mustafa Kareem Jawad, Fatimah Saleh Alsuwayidi, and Ameer Hasan Kadhem. Formal analysis: Wahby Mohammed Ahmed Babaresh, Salim K. Hajwal, Hasan Naeem Kareem, and Abdur Rahim. Investigation: Alaa Hamza Hermis, Sajjad Sadeq Salman, Murtadha Abdulridha Ajel, and Fadhilah N. Alobaidan. Methodology: Asif Jan, Wahby Mohammed Ahmed Babaresh, Hasan Naeem Kareem, and Waheed Ali Shah. Project administration: Aftab Ullah, Waheed Ali Shah, Abdur Rahim, and Wahby Mohammed Ahmed Babaresh. Resources: Alaa Hamza Hermis, Mustafa Kareem Jawad, Fatimah Saleh Alsuwayidi, and Ameer Hasan Kadhem. Software: Salim K. Hajwal, Sajjad Sadeq Salman, Murtadha Abdulridha Ajel, and Syed Shaukat Ali. Supervision: Wahby Mohammed Ahmed Babaresh, Asif Jan, Hasan Naeem Kareem, and Waheed Ali Shah. Validation: Abdur Rahim, Syed Shaukat Ali, Fadhilah N. Alobaidan, and Ameer Hasan Kadhem. Visualization: Fatimah Saleh Alsuwayidi, Mustafa Kareem Jawad, Salim K. Hajwal, and Sajjad Sadeq Salman. Writing – original draft: Aftab Ullah, Fatimah Saleh Alsuwayidi, Murtadha Abdulridha Ajel, and Syed Shaukat Ali. Writing – review and editing: all authors approved the final version to submit.

## Ethics Statement

The authors have nothing to report as there were no human participants involved in this study.

## Consent

The authors have nothing to report as this study does not involve any individual person's data in any form.

## Conflicts of Interest

The authors declare no conflicts of interest.

## Data Availability

All data generated or analyzed during this study are included in this published article (and its Supporting Information files).

## References

[1] M. G. Saklayen , “The Global Epidemic of the Metabolic Syndrome,” Current Hypertension Reports 20, no. 2 (2018): 12. 29480368 10.1007/s11906-018-0812-z PMC5866840

[2] F. Montazerifar , A. Bolouri , M. M. Mozaffar , and M. Karajibani , “The Prevalence of Metabolic Syndrome in Coronary Artery Disease Patients,” Cardiology Research 7, no. 6 (2016): 202–208. 28197293 10.14740/cr507w PMC5295511

[3] L. L. Demer and Y. Tintut , “Vascular Calcification: Pathobiology of a Multifaceted Disease,” Circulation 117, no. 22 (2008): 2938–2948. 18519861 10.1161/CIRCULATIONAHA.107.743161 PMC4431628

[4] P. Greenland , M. J. Blaha , M. J. Budoff , R. Erbel , and K. E. Watson , “Coronary Calcium Score and Cardiovascular Risk,” Journal of the American College of Cardiology 72, no. 4 (2018): 434–447. 30025580 10.1016/j.jacc.2018.05.027 PMC6056023

[5] A. Devesa , V. Fuster , R. Vazirani , et al., “Cardiac Insulin Resistance in Subjects With Metabolic Syndrome Traits and Early Subclinical Atherosclerosis,” Diabetes Care 46, no. 11 (2023): 2050–2057. 37713581 10.2337/dc23-0871 PMC10632182

[6] S. M. Riahi , A. Fanoodi , S. Shetty , and S. S. Hashemi‐Nazari , “Critical Assessment of the Metabolic Syndrome Definitions in the Adult General Population of the United States‐The Multi‐Ethnic Study of Atherosclerosis (MESA),” Journal of Diabetes and Metabolic Disorders 22, no. 1 (2023): 851–859. 37255828 10.1007/s40200-023-01213-5 PMC10225431

[7] I. Garba , P. Engel‐Hills , F. Davidson , and A. Ismail , “Radiation Dose Management System in Computed Tomography Procedures: A Systematic Review,” Radiation Protection Dosimetry 199, no. 10 (2023): 1063–1074. 37078550 10.1093/rpd/ncad124

[8] M. J. Page , J. E. McKenzie , P. M. Bossuyt , et al., “The PRISMA 2020 Statement: An Updated Guideline for Reporting Systematic Reviews,” BMJ 372 (2021): n71. 33782057 10.1136/bmj.n71 PMC8005924

[9] Z. Munn , S. Moola , D. Riitano , and K. Lisy , “The Development of a Critical Appraisal Tool for Use in Systematic Reviews Addressing Questions of Prevalence,” International Journal of Health Policy and Management 3, no. 3 (2014): 123–128. 25197676 10.15171/ijhpm.2014.71 PMC4154549

[10] M. Shabil , G. Bushi , and M. N. Khatib , “A Commentary on “Psychological Health Among Healthcare Professionals During COVID‐19 Pandemic: An Updated Meta‐Analysis”,” Indian Journal of Psychiatry 66, no. 8 (2024): 763–764. 39398520 10.4103/indianjpsychiatry.indianjpsychiatry_496_24 PMC11469564

[11] T. Alrahbeni , A. Mahal , A. Alkhouri , et al., “Surgical Interventions for Intractable Migraine: A Systematic Review and Meta‐Analysis,” International Journal of Surgery 110, no. 10 (2024): 6306–6313. 38626410 10.1097/JS9.0000000000001480 PMC11486983

[12] G. Bushi , B. K. Padhi , M. Shabil , et al., “Cardiovascular Disease Outcomes Associated With Obstructive Sleep Apnea in Diabetics: A Systematic Review and Meta‐Analysis,” Diseases 11, no. 3 (2023): 103. 37606474 10.3390/diseases11030103 PMC10443251

[13] M. Shabil , G. Bushi , P. K. Bodige , et al., “Effect of Fenugreek on Hyperglycemia: A Systematic Review and Meta‐Analysis,” Medicina 59, no. 2 (2023): 248. 36837450 10.3390/medicina59020248 PMC9962665

[14] M. Shabil , G. Bushi , N. Rai , and H. Abu Serhan , “Comment on: “Efficacy and Safety of Tebentafusp in Patients With Metastatic Uveal Melanoma: A Systematic Review and Meta‐Analysis”,” Human Vaccines & Immunotherapeutics 20, no. 1 (2024): 2398870. 39431660 10.1080/21645515.2024.2398870 PMC11500552

[15] M. Shabil , A. Yadav , M. A. Shamim , et al., “Prevalence of Hepatitis B and C Infections Among HIV‐Positive Men Who Have Sex With Men: A Systematic Review and Meta‐Analysis,” Health Science Reports 7, no. 6 (2024): e2206. 38933421 10.1002/hsr2.2206 PMC11199987

[16] M. Shabil , G. Bushi , M. A. Beig , M. A. Rais , M. Ahmed , and B. K. Padhi , “Cardiovascular Manifestation in Tuberculosis Cases: A Systematic Review and Meta‐Analysis,” Current Problems in Cardiology 48, no. 7 (2023): 101666. 36828041 10.1016/j.cpcardiol.2023.101666

[17] I. J. Kullo , A. E. Cassidy , P. A. Peyser , S. T. Turner , P. F. Sheedy , and L. F. Bielak , “Association Between Metabolic Syndrome and Subclinical Coronary Atherosclerosis in Asymptomatic Adults,” American Journal of Cardiology 94, no. 12 (2004): 1554–1558. 15589016 10.1016/j.amjcard.2004.08.038

[18] S. Masrouri , M. D. Shapiro , D. Khalili , and F. Hadaegh , “Impact of Coronary Artery Calcium on Mortality and Cardiovascular Events in Metabolic Syndrome and Diabetes Among Younger Adults,” European Journal of Preventive Cardiology 31, no. 6 (2024): 744–753. 38323650 10.1093/eurjpc/zwae039

[19] R. D. Santos , K. Nasir , K. Tufail , R. S. Meneghelo , J. A. M. Carvalho , and R. S. Blumenthal , “Metabolic Syndrome Is Associated With Coronary Artery Calcium in Asymptomatic White Brazilian Men Considered Low‐Risk by Framingham Risk Score,” Preventive Cardiology 10, no. 3 (2007): 141–146. 17617777 10.1111/j.1520-037x.2007.888128.x

[20] N. D. Wong , J. C. Nelson , T. Granston , et al., “Metabolic Syndrome, Diabetes, and Incidence and Progression of Coronary Calcium,” JACC: Cardiovascular Imaging 5, no. 4 (2012): 358–366. 22498324 10.1016/j.jcmg.2011.12.015 PMC3327555

[21] R. C. Ellison , Y. Zhang , L. E. Wagenknecht , et al., “Relation of the Metabolic Syndrome to Calcified Atherosclerotic Plaque in the Coronary Arteries and Aorta,” American Journal of Cardiology 95, no. 10 (2005): 1180–1186. 15877990 10.1016/j.amjcard.2005.01.046

[22] N. D. Wong , H. Gransar , L. J. Shaw , D. Polk , and D. S. Berman , “Comparison of Atherosclerotic Calcification Burden in Persons With the Cardiometabolic Syndrome and Diabetes,” Journal of the Cardiometabolic Syndrome 1, no. 2 (2006): 90–94. 17679823 10.1111/j.1559-4564.2006.05618.x

[23] U. N. Ibebuogu , N. Ahmadi , F. Hajsadeghi , et al., “Measures of Coronary Artery Calcification and Association With the Metabolic Syndrome and Diabetes,” Journal of the Cardiometabolic Syndrome 4, no. 1 (2009): 6–11. 19245510 10.1111/j.1559-4572.2008.00028.x

[24] H. Cao , X. Chen , J. Lu , et al., “Metabolic Syndrome and Coronary Artery Calcification: A Community‐Based Natural Population Study,” Chinese Medical Journal 126, no. 24 (2013): 4618–4623. 24342299

[25] M. H. Seo , E.‐J. Rhee , S. E. Park , et al., “Metabolic Syndrome Criteria as Predictors of Subclinical Atherosclerosis Based on the Coronary Calcium Score,” Korean Journal of Internal Medicine 30, no. 1 (2015): 73. 25589838 10.3904/kjim.2015.30.1.73 PMC4293567

[26] M.‐K. Lee , H.‐J. Park , W. S. Jeon , et al., “Higher Association of Coronary Artery Calcification With Non‐Alcoholic Fatty Liver Disease Than With Abdominal Obesity in Middle‐Aged Korean Men: The Kangbuk Samsung Health Study,” Cardiovascular Diabetology 14 (2015): 88. 26169265 10.1186/s12933-015-0253-9 PMC4501081

[27] S. Y. Lee , Y. Y. Hyun , K. B. Lee , and H. Kim , “Low Serum Magnesium Is Associated With Coronary Artery Calcification in a Korean Population at Low Risk for Cardiovascular Disease,” Nutrition, Metabolism, and Cardiovascular Diseases 25, no. 11 (2015): 1056–1061. 10.1016/j.numecd.2015.07.010 26472514

[28] M. K. Kim , C. W. Ahn , S. Kang , J. S. Nam , K. R. Kim , and J. S. Park , “Relationship Between the Triglyceride Glucose Index and Coronary Artery Calcification in Korean Adults,” Cardiovascular Diabetology 16 (2017): 108. 28830471 10.1186/s12933-017-0589-4 PMC5568209

[29] S. Malik , Y. Zhao , M. Budoff , et al., “Coronary Artery Calcium Score for Long‐Term Risk Classification in Individuals With Type 2 Diabetes and Metabolic Syndrome From the Multi‐Ethnic Study of Atherosclerosis,” JAMA Cardiology 2, no. 12 (2017): 1332–1340. 29117273 10.1001/jamacardio.2017.4191 PMC5814996

[30] E. Ekblom‐Bak , Ö. Ekblom , E. Fagman , et al., “Fitness Attenuates the Prevalence of Increased Coronary Artery Calcium in Individuals With Metabolic Syndrome,” European Journal of Preventive Cardiology 25, no. 3 (2018): 309–316. 29171773 10.1177/2047487317745177

[31] M. Marjanovic Petkovic , M. Vuksanovic , D. Sagic , I. Radovic , I. Soldatovic , and T. Beljic Zivkovic , “Risk Factors for Coronary Artery Calcifications in Overweight or Obese Persons With Prediabetes: Can They Predict T2 Diabetes and Coronary Vascular Events?,” Journal of Clinical Medicine 12, no. 12 (2023): 3915. 37373609 10.3390/jcm12123915 PMC10299489

[32] H. Fakhry , M. Abo Elsoud , M. Abdel Raheem , and K. Abdel Wahab , “Coronary Calcium in Patients With Metabolic Syndrome: Presence and Extent by MSCT,” Ain Shams Medical Journal 74, no. 1 (2023): 97–109.

[33] S. Tangjitgamol , W. Udayachalerm , P. Preeyanont , W. Kaewwanna , N. Ativanichayapong , and C. Wanishsawad , “Metabolic Syndrome and the Risk of Coronary Artery Disease Among the Physicians,” Annals of Medicine & Surgery 86, no. 2 (2024): 761–767. 38333252 10.1097/MS9.0000000000001630 PMC10849357

